# Effect of Strain Rate Sensitivity on Fracture of Laminated Rings under Dynamic Compressive Loading

**DOI:** 10.3390/ma15020472

**Published:** 2022-01-08

**Authors:** Amir Partovi, Mohammad Mehdi Shahzamanian, Peidong Wu

**Affiliations:** 1 Department of Mechanical Engineering, McMaster University, Hamilton, ON L8S 4L7, Canada; peidong@mcmaster.ca; 2 Department of Civil and Environmental Engineering, University of Alberta, Edmonton, AB T6G 2W2, Canada; mshahzam@ualberta.ca

**Keywords:** ring compression test, strain rate sensitivity, cladding, topological arrangement, finite element method (FEM)

## Abstract

The effects of cladding layers of rate-sensitive materials on the ductility and fracture strain of compressed rings are numerically investigated by using the finite element method (FEM) and employing the Johnson–Cook (J–C) model. The results show that ductility is governed by the behavior of the material that is located at the ring outer wall regardless of the volume fraction of the core and clad materials. However, as the number of layers increases, this influence becomes less noticeable. Moreover, as barreling increases at the outer wall and decreases at the inner wall, fracture strain increases. Furthermore, the effects of ring shape factor and bonding type of clad and core materials are numerically evaluated. The numerical results show that less force per unit volume is required to fracture narrower rings and that using a noise diffusion pattern at the interface of the materials is more suitable to simulate crack propagation in the compressed rings and functionally graded materials (FGMs). Additionally, delamination has a direct relation to layer thickness and can occur even in the presence of perfect bonding conditions owing to differences among the material and fracture parameters of laminated layers.

## 1. Introduction

Cladding is a technique for bonding the layers of different materials together to enhance the characteristics of the core material. Depending on the industrial application of the components, improvements can be made in various ways [1,2,3]. In mechanical engineering applications, this process is primarily performed to increase the ductility of base materials [4]. Strain rate sensitivity, shear band formation, and crack propagation are some important features that must be considered in dynamic loading applications [5]. In this work, the effects of cladding strain rate-sensitive and rate-insensitive layers on the ductility and fracture of rings under axial compressive loading are numerically investigated via finite element analysis (FEA).

Advanced fabrication processes, such as additive manufacturing (AM), enable researchers to consider and evaluate structures made of different materials with complicated shapes, thereby expanding the applications of simulations and FEA. The laser cladding method is based on the fundamentals of directed energy deposition processes in AM technology. By capitalizing on AM processes, including 3D printers, engineers and researchers have been able to design complex structures during the last few decades. The AM technique, especially the laser-based powder bed fusion, has become the focal point of various research endeavors and is constantly advancing [6,7]. Currently, it is possible to manufacture irregular components made of engineering materials that adequately bond together [8]. The development of these techniques enables researchers to investigate advancements and promote new objectives requiring complicated shapes with a relatively complicated material pattern by utilizing FEA. This endeavor is the aim of the present study.

Analyzing the effects of cladding and coating on different materials under different loading conditions is widely conducted. Two examples are analyses of the ring under radial rapid internal pressure and the ring under lateral compression [9,10]. Shi et al. [11] numerically investigated the necking phenomenon in tubes with the layers of different materials subjected to high-speed loading. The constitutive model in their numerical analysis was introduced by Weber and Anand [12]. They performed plane strain analysis and considered that the rate-insensitive core material, AA6111, was coated from both sides with a rate-dependent material. Their simulations showed the significant effect of ductile material and the ratio of cladded to core material on delaying necking initiation. 

Hu et al. [13] determined the effects of applying layers of a rate-sensitive material on the core material that was considered to be rate-insensitive and observed the necking phenomenon in the cladded sheets. Both core and clad materials in their analysis were defined by the power law. Their numerical simulations showed that the increase in strain rate sensitivity and the volume fraction of ductile coating material resulted in the retardation of necking. They also observed an increase in the necking strain as the volume fraction of the ductile material increased. The numerical investigations of Chen et al. [14] on a cladded sheet under tension showed that the contribution of ductile base material improved necking strain and delayed fracture. Their results also showed the influence of mesh size on the fracture of the specimens. One of the main issues in the layered structures occurs at the interface of the two different materials and is related to the debonding phenomenon. Lonetti [15] proposed a model to predict the dynamic propagation of interfacial cracks. The proposed model was based on the fundamentals of fracture mechanics and also moving mesh techniques. Funari et al. [16] continued this work by introducing a methodology to find the location of crack initiation and also the crack propagation path. 

The necking phenomenon in a ductile metal is the starting point towards fracture [11]. The necking behavior in a tensile test is similar to the barreling behavior in a compression test. Barreling, which is the consequence of friction between the die and workpiece, occurs when a test coupon under compression undergoes plastic deformation [17,18,19]. Causing a delay in necking can hinder the occurrence of a fracture and consequently enhance the ductility of the material under compression. Therefore, by employing some practical techniques, such as applying hydrostatic pressure on the specimen or coating a ductile material on the base material, and by controlling stress, the triaxiality, barreling and fracture of the material can be postponed [20,21]. When a ring is compressed and its height is decreased, the material can flow radially towards inner and outer surfaces. In general, in the case of low friction between the ring and the platens, the material flows outwards, and in the presence of high friction, the material at the inner wall flows inwards, and the inner diameter decreases [17,18,19]. Barreling occurs when the friction is high, and the ring walls start to bulge. This phenomenon results in the likelihood of starting and growing a crack from the side surfaces that needs to be evaluated when forming or forging bulk metals.

In this study, a numerical simulation of a fracture in a compressed ring is performed by an FE model in the commercial software ABAQUS. Some of the constitutive models to numerically evaluate large non-reversible deformation and fracture of ductile components under different loading situations are the Gurson–Tvergaard–Needleman (GTN), power law and Johnson–Cook (J–C) models [22,23,24]. In this work, because of the shear fracture mode in the compressed rings and considering the objectives to investigate the behavior of samples under different applied loads, the J–C model is utilized. The J–C constitutive law includes stress triaxiality and depends on strain rate in the material fracture model. Wierzbicki et al. [25] demonstrated that the J–C model can be utilized for the evaluation of ductile fracture when the stress triaxiality has a known interval and is limited. When a ring is under compressive loading, the stress triaxiality will have negative values that are the consequence of compressive stress states in the material. This is the natural behavior expected in a ring compression test. Estimation and calibration of parameters used in the J–C material and fracture models for bulk metal forming and cutting applications have been the focus of numerous researchers [26,27,28].

The focus of the current study is on applying layers of clad materials to the side walls of a compressed ring. This work numerically investigates how the topography of layers of rate-insensitive and rate-sensitive materials (such as base and clad materials) influences the deformation and failure of rings under dynamic compressive loading. This analysis is performed by changing the number and location of layers. The effect of geometry on the failure strain of cladded rings subjected to dynamic compressive loading is also numerically investigated. After performing these simulations and finding the general trends in the f–d curves and crack initiation patterns, some parametric studies, including the effect of volume fraction and shape factor, will be developed. Finally, the effect of having layers of brittle and soft materials in the ring will be briefly investigated. To the best of our knowledge, the effects of cladding strain rate-sensitive layers on the failure and compressibility of metallic rings have not been considered numerically in detail elsewhere.

## 2. Constitutive Model

The summation of the stress deviator tensor ($s_{ij}$) and hydrostatic stress tensor ($\sigma_{m}\delta_{ij}$), or the mean normal stress tensor, results in the true stress tensor as follows:

(1) $$\sigma_{ij}=s_{ij}+\sigma_{m}\delta_{ij}.\;$$

In the above equation, $\sigma_{ij}$ indicates the components of the true stress tensor, and $\delta_{ij}$ is the unit matrix, whose dimensions are similar to that of the stress tensor. The hydrostatic stress, $\sigma_{m}$, can be expressed as:

(2) $$\sigma_{m}=\frac{\sigma_{11}+\sigma_{22}+\sigma_{33}}{3}.\;$$

The mean stress tensor is responsible for the volume change in a body that is under stress and governs the void growth. On the other hand, the stress deviator tensor governs the change in the shape or distortion [29]. Stress triaxiality is defined as the ratio of mean to equivalent stress as follows:

(3) $$\eta =\frac{\sigma_{m}}{\sigma_{eq}}.\;$$

The effect of rate sensitivity on the compressed ring is numerically investigated in ABAQUS using the J–C material model. The J–C constitutive law is defined as follows [30]:

(4) $$\sigma_{eq}=\left[A+B\varepsilon_{P}^{n}\right]\left[1+C\ln\left(\frac{{\dot{\varepsilon }}_{P}}{{\dot{\varepsilon }}_{0}}\right)\right]\left[1-{\left(\frac{T-T_{0}}{T_{m}-T_{0}}\right)}^{m}\right],\;$$

where $\sigma_{eq}$ is the equivalent stress, $\varepsilon_{P}$ is the equivalent plastic strain, ${\dot{\varepsilon }}_{p}$ is the equivalent plastic strain rate and ${\dot{\varepsilon }}_{0}$ is the reference strain rate. The constant material parameters are *A*, *B*, *C*, *n* and *m*. In Equation (4), *T* is the operating temperature, $T_{0}$ is the room temperature and $T_{m}$ indicates the melting temperature of the material. 

The J–C fracture model is defined as:

(5) $$\varepsilon_{f}=\left[D_{1}+D_{2}Exp\left(D_{3}\eta \right)\right]\left[1+D_{4}\ln\left({\dot{\varepsilon }}_{P}^{*}\right)\right]\left[1+D_{5}T^{*}\right],\;$$

where $\varepsilon_{f}$ is the fracture strain, and ${\dot{\varepsilon }}_{P}^{*}$ is the normalized plastic strain rate by the reference strain rate of 1.0 1/s. The material constants are $D_{1}$ to $D_{5}$, and η denotes the stress triaxiality. In Equation (5), $T^{*}$ is the homologous temperature. To evaluate stress triaxiality, the sign of $D_{3}$ must be changed in the material settings in ABAQUS [28,31].

**Figure 1:**
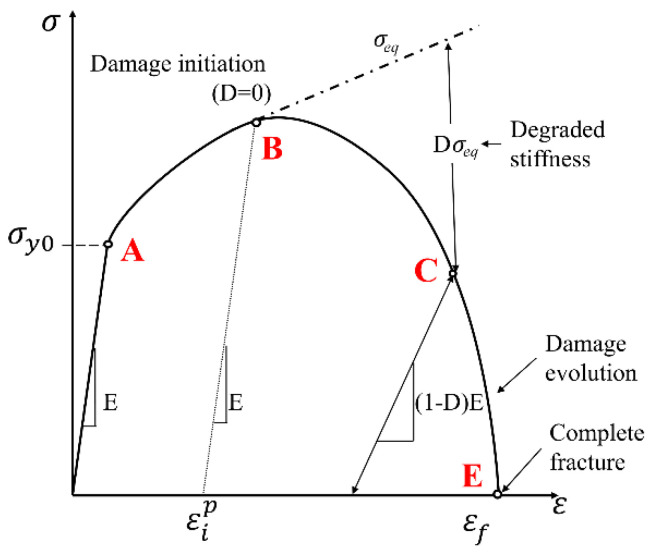
Schematic representation of damage evolution in the software on a typical stress–strain curve [32,33].

The initiation of crack is simulated in ABAQUS/Explicit using the element deletion method. The damage rule and parameter is calculated according to a linear accumulative criterion which is defined as:

(6) $$D=\sum^{\;}\frac{\Delta \varepsilon }{\varepsilon_{f}}\leq 1,\;$$

where Δε is the increment of the effective plastic strain, and $\varepsilon_{f}$ denotes the strain at failure. The damage parameter (*D*) changes from 0 and 1, where 0 indicates the non-damaged material, and 1 indicates material failure. At any time increment throughout the simulation, the damaged stress state is evaluated by

(7) $$\sigma_{D}=\left(1-D\right)\sigma_{eq},$$

where *D* is the damage value or parameter, and $\sigma_{eq}$ is the equivalent stress obtained from the present time increment. Figure 1 shows the process of damage evolution in the software on a typical stress–strain curve that is shown by the solid line [32,33]. The dashed line represents the undamaged stress–strain curve. Point A denotes the yield stress with no plastic strain. The damage initiates from point B, after which the stress and modulus of elasticity decrease as the effective plastic strain increases. When the critical value of the damage parameter, which is unity, is reached, material failure occurs in the software, and the corresponding element is deleted from the meshed structure (point E). 

## 3. Problem Formulation and Method of Solution

**Figure 2:**
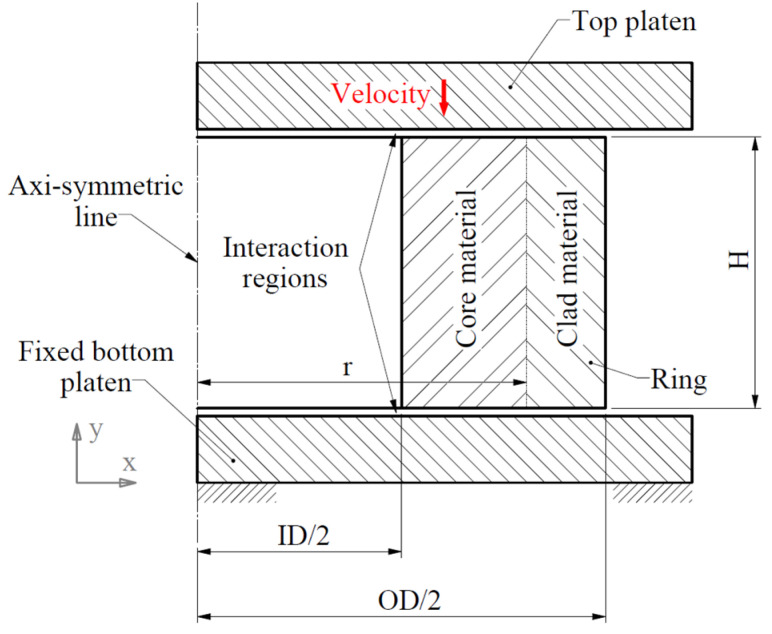
Schematic representation: the ring with laminated layers under dynamic compressive loading.

**Figure 3:**
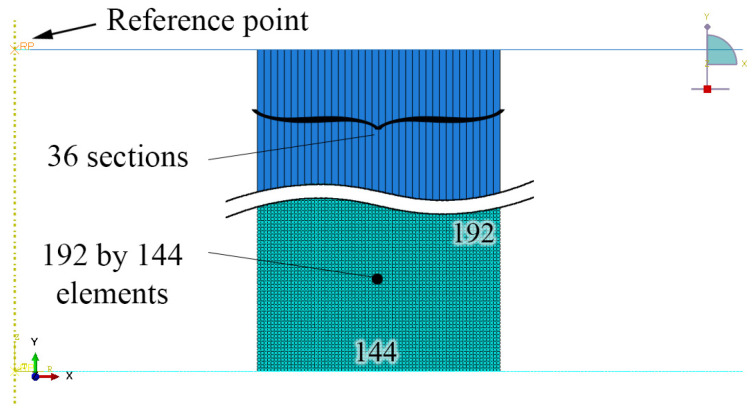
FE configuration of the ring model in ABAQUS, with the RP of the top platen where the initial velocity is applied.

Figure 2 depicts the schematic of the ring with laminated layers under compressive loading. The FE configuration is defined in a 2D axisymmetric model in ABAQUS/Explicit. Given that the fracture mode is shear, symmetry in the x-direction cannot be applied. ABAQUS/Explicit is utilized to accommodate the J–C fracture model. The following descriptions of the schematic of the ring are provided in Ref. [20]. In brief, the ring has an outer diameter (OD) of 18 mm, inner diameter (ID) of 9 mm, and height (H) of 6 mm, which is in accordance with a ratio of 6:3:2 (OD:ID:H) that is known as the standard ratio for the geometry. The element type of RAX2 is considered for the rigid bottom platen that has a fixed reference point (RP). The ring is modeled with CAX4R (4-node linear axisymmetric) elements considering reduced integration with hourglass control. The element type of RAX2 is selected for the rigid top platen. A constant velocity is applied on the RP of the top platen to move towards down and compress the ring model. Figure 3 shows the FE configuration of the model in ABAQUS. The cross-section of the ring is divided vertically into 36 sections for applying different material properties. Each section contains 192 by 4 elements; hence, the ring has a total of 27,648 elements. A small gap of 0.001 mm (=element size/32) is placed between the platens and the specimen to reduce the effect of the impact and avoid a sudden and sharp increase at the beginning of the force–displacement (f–d) curve at high speeds. The coefficient of friction is set to be 0.2 in the contact property in the software.

## 4. Results and Discussions

The material parameters of the J–C model utilized in this work are shown in Table 1 [34]. The rate sensitivity parameter, *C*, will be changed to control the strain rate dependency of the material. Table 2 presents the failure parameters of the J–C model of this material [34]. It should be emphasized that the key objective of the current work is to evaluate the effects of laminated layers of rate-sensitive and rate-insensitive materials on the compressibility of metallic rings and that the presented results and the overall conclusions do not specifically depend on the applied values of the material parameters.

**Figure 4:**
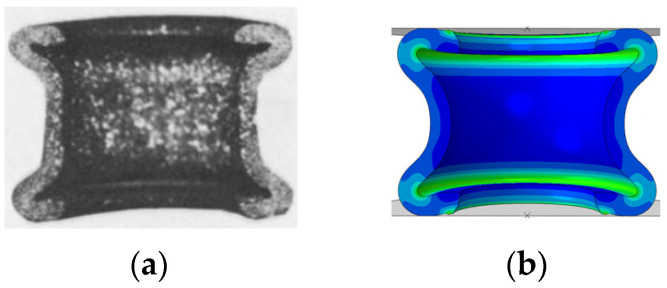
Comparison of (**a**) the result of the experiment [35] with (**b**) the result of the simulation for the verification of procedure in ABAQUS.

Mesh sensitivity analysis is performed by changing the size of the elements’ aspect ratio. As reported in Ref. [20], increasing the element ratio (height/width ratio > 1) does not lead to a proper deformation pattern of under compression rings. Therefore, square elements are considered in this study. The configuration setup in ABAQUS is validated by comparing the simulation of a model and an existing result from the experiment [35] (Figure 4). The simulation of the fracture mechanism of monolithic material rings was verified in a previous work [20], where the numerical results were validated with the experimental results of Gang et al. [36].

### 4.1. Effects of Number and Location of Layers

**Figure 5:**
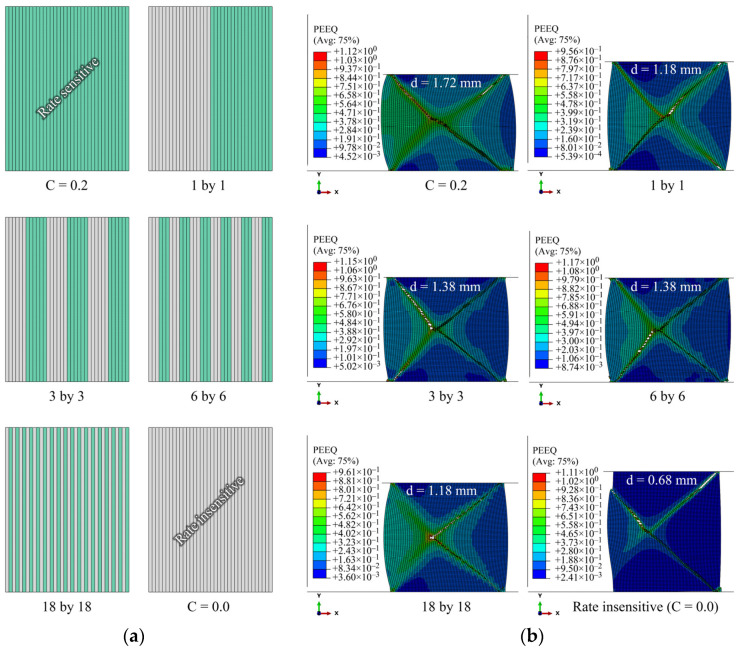
(**a**) Topographic profile of laminated layers in the cross-section of the ring, (**b**) effect of laminated layers on the fracture of the rings. The displacement level, *d*, at which the distribution of PEEQ is presented, is shown above each image.

**Figure 6:**
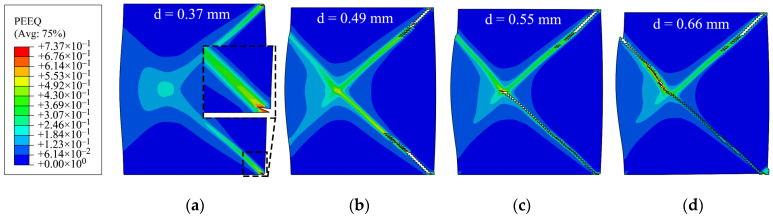
Sequence of crack propagation in a typical compressed ring specimen: (**a**) formation of shear bands and crack initiation at the corner of outer wall; (**b**) crack propagates along the shear bands; (**c**) crack reaches the center of the cross-section; and (**d**) crack reaches the inner wall (complete rupture). The stroke of the top platen, *d*, is shown above the models.

**Figure 7:**
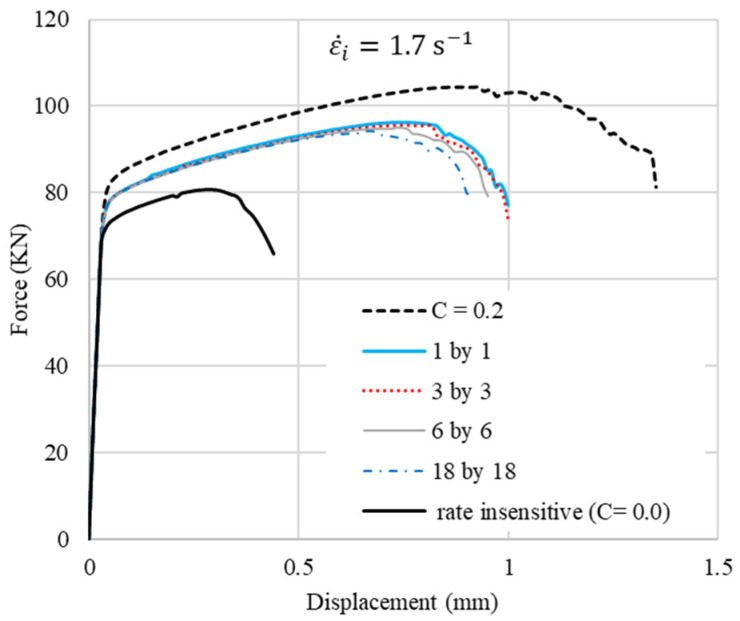
Effect of cladding on f–d curves.

Given that the model has 36 vertical material sections, six different laminated cases, 1by1 to 18by18 layers, are considered. Rate insensitive layers and rate-sensitive layers are arranged alternatively from left (inner wall) to right (outer wall), starting with a rate-insensitive layer. The results are shown in Figure 4. The left edge represents the inner radius of the ring, as illustrated in the FE configuration in Figure 3. The force–displacement (f–d) curves of the rings are shown in Figure 5. The ductility of the ring increases by an increase in the strain rate sensitivity of the material. It should be noted that in all these laminated cases, the rate-sensitive material is located at the outer wall. Regardless of the number of layers, cracks start from the corner of the outer walls along the shear bands with an angle of about 45° between them and the top or bottom platen. The f–d curves are very similar for the four laminated cases. However, as the number of layers decreases, crack initiation is delayed. In other words, the more rate-sensitive material at the outer wall, where the crack starts, the more ductility is gained. The volume fractions of rate-insensitive to rate-sensitive materials for the four cases of 1by1 to 18by18 models in Figure 5 are approximately 42–58%, 47–53%, 49–51% and 50–50%, respectively. The stroke of top platen or displacement level, *d*, at which the distribution of equivalent plastic strain is presented, is indicated above each image in Figure 5b. It should be noted that the criterion for assessing ductility in this work is the initiation of the crack, not the complete rupture of the models. The sequence of crack initiation and propagation in a compressed ring, for the case of pure rate-insensitive material, is illustrated in Figure 6. The force–displacement curve of this case is shown in Figure 7 by the solid black line. In Figure 7, also in the following figures, 1.7/s is the initial applied strain rate. During the deformation process, the applied strain rate monotonically increases. This is due to the fact that the height of a ring decreases, while the impact velocity is constant.

**Figure 8:**
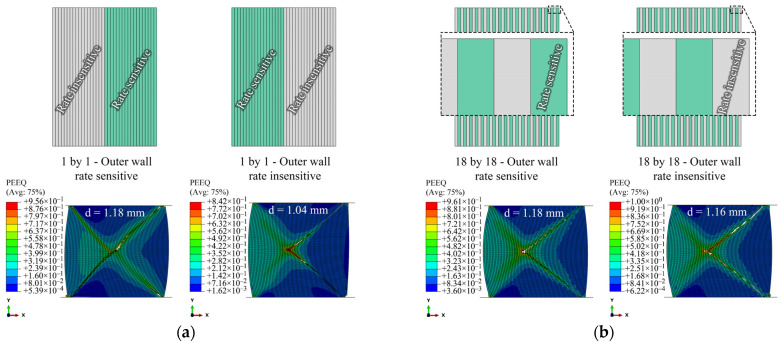
(**a**) 1by1 and (**b**) 18by18 models; rate-sensitive and insensitive at inner and outer walls. The stroke of the top platen, *d*, is shown above the models.

**Figure 9:**
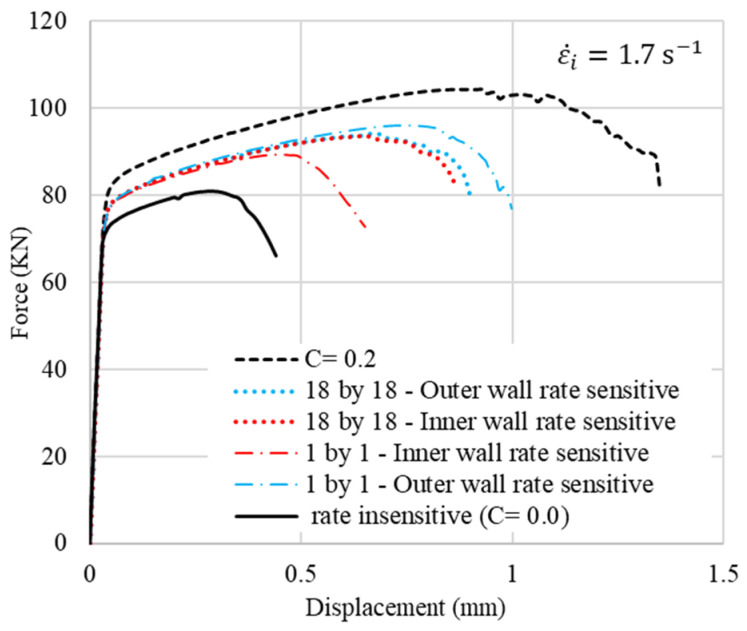
F–d curves of 1by1 and 18by18 models.

The effects of the location of the layers are evaluated using two different models, namely 1by1 and 18by18 models with rate-sensitive and rate-insensitive materials located at the inner and outer walls, as shown in Figure 8a,b, respectively. The results of the f–d curves are shown in Figure 9. The f–d curves of 1by1 models have a considerable difference when the rate-sensitive material is located either inside or outside. However, this difference is less remarkable in the case of 18by18 models. A reason that may account for this behavior could be the difference in the volume fraction of the materials in the 1by1 models. This difference is investigated in the next section.

After crack initiation, the models with a smaller number of layers reach the fully fractured point more quickly than the models with a larger number of layers. Thus, the time is shorter from the first element deletion (crack initiation) to the full separation of elements (fully ruptured model). Here, owing to inhomogeneity, each interface line can act as a barrier and consequently makes more delays for crack propagation along the shear bands. This supposition is in agreement with the experimental results of Syn et al. [37]. They experimentally observed that, in the case of laminated layers under tension, the increase in the number of laminated layers results in a more tortuous path for crack propagation.

In the present work, it is assumed that there is a perfect bonding condition between the layers. The direction of the crack propagation is highly related to the formation and direction of shear bands. Since there is no imperfection inside the models (e.g., to simulate cavity), there is no geometrical trigger or defect inside the model to be able to change the direction of the crack propagation rather than the shear bands. However, the effects of inhomogeneity on crack propagation path will be studied briefly in the last section of the results considering layers of ductile and brittle materials.

### 4.2. Effects of Volume Fraction

**Figure 10:**
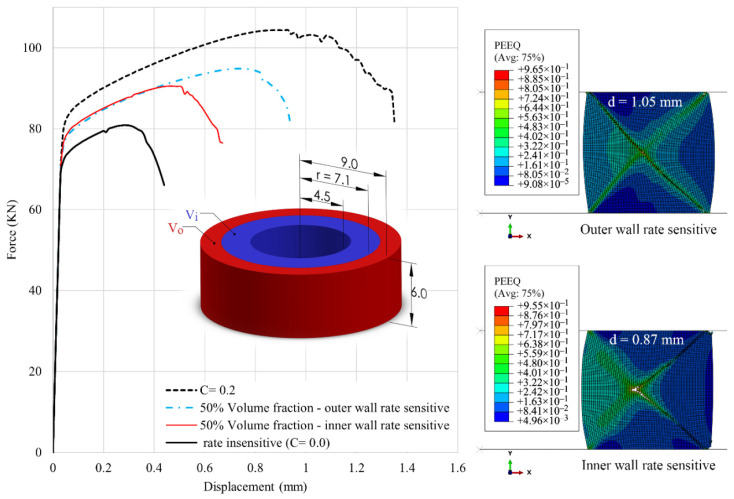
Effect of location of rate-sensitive material with 50% volume fraction on the f–d curves. On the right, the distribution of equivalent plastic strain is shown for the case of outer wall rate-sensitive at the displacement level of d=1.05 mm, and for the case of inner wall rate-sensitive at the displacement level of d=0.87 mm.

The effects of volume fraction are further investigated by assuming that the rate-sensitive and the rate-insensitive materials have the same (50%) volume fractions, with a partition line located in the ABAQUS model at r = 7.12 mm. In this case, the volume of the inner material (*V_i_*) is equal to the volume of the outer material (*V_o_*). The results are shown in Figure 10. Without considering the fracture, the f–d curves of the 50% cladded models are almost halfway between those of the pure materials. However, the crack initiation strain largely depends on the location of the rate-sensitive material and increases by approximately 60% when this material is located at the outer wall. Therefore, the ductility of a ring under compression is governed not only by the volume fractions but also by the topographic arrangement of layers.

**Figure 11:**
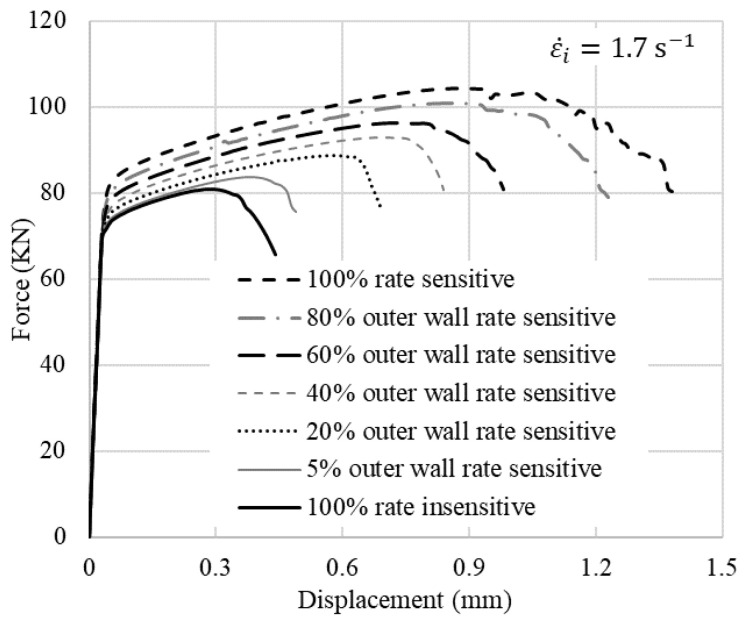
Effect of different volume fractions on the f–d curves.

**Figure 12:**
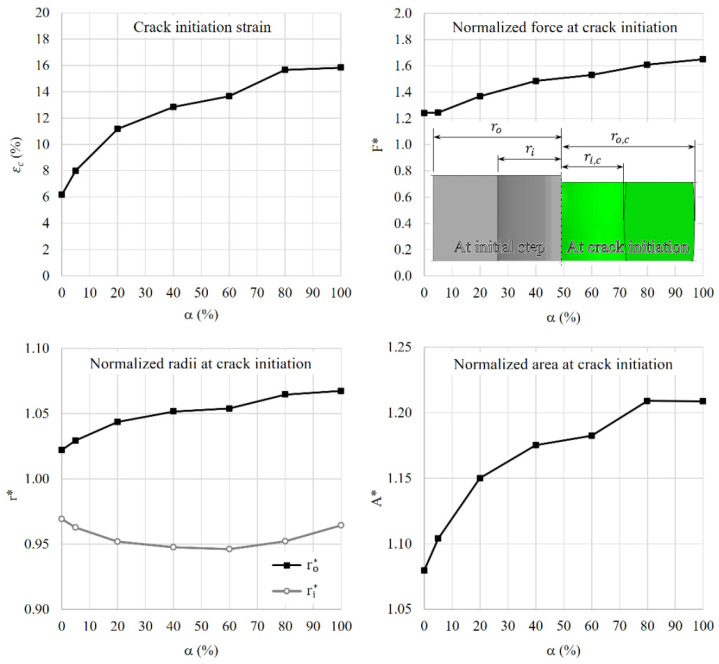
Effect cladding ratio on overall response of the rings.

The same process as above is followed to study the effects of cladding from the outer wall with different volume fractions of rate-sensitive material, α. The ductility of the ring is measured in terms of the crack initiation strain, which is calculated as:

(8) $$\varepsilon_{c}=\frac{H_{i}-H_{c}}{H_{i}}\times 100\%,\;$$

where $H_{i}$ is the initial height of the ring, and $H_{c}$ is the height at which crack initiates. Following Chen et al. [14], the overall behavior of the compressed cladded rings can be compared in terms of normalized force F*, normalized maximum cross-sectional area A* and normalized radii r* as follows:

(9) $$F^{*}=\frac{F_{c}}{\sigma_{y}^{core}A_{i}^{core}+\sigma_{y}^{clad}A_{i}^{clad}}=\frac{F_{c}}{\sigma_{y}A_{i}}\;$$

(10) $$A^{*}=\frac{A_{max}}{A_{i}}=\frac{A_{c}}{A_{i}},A_{c}=\pi \left(r_{o,c}^{2}-r_{i,c}^{2}\right)$$

(11) $$r_{i}^{*}=\frac{r_{i,c}}{r_{i}}\left(r_{o}^{*}=\frac{r_{o,c}}{r_{o}}\right)$$

where *F_c_*, *A_c_*, *r_i,c_* and *r_o,c_* are the applied force, maximum cross-sectional area, inner radius and outer radius at the onset of crack initiation, respectively. *A_i_* is the initial cross-sectional area. The results are presented in Figure 11 and Figure 12. The f–d curves linearly increase as the contribution of the rate-sensitive material to the model increases. The normalized crack initiation force shows an overall linear relation with the cladding ratio, α, which is the ratio between the volume of the cladding material to the total volume of the ring. However, 5% of the rate-sensitive material at the outer wall does not considerably change the flow stress but delays the displacement at fracture by about 25%. The rate of change in the normalized area and strain at fracture decreases as the cladding ratio increases.

A coefficient of friction of 0.2 is defined in the contact property of the ring and the two platens in the software. The use of this coefficient of friction results in an outward flow of material at the outer surface and an inward flow in the inner surface. The outer radius constantly increases with the increase in the cladding ratio. However, the inner radius decreases up to the cladding ratio of 60%. Subsequently, the rate of reduction in the inner radius is lessened so that the dimension of the inner radius at crack initiation for the monolithic materials becomes almost the same.

**Figure 13:**
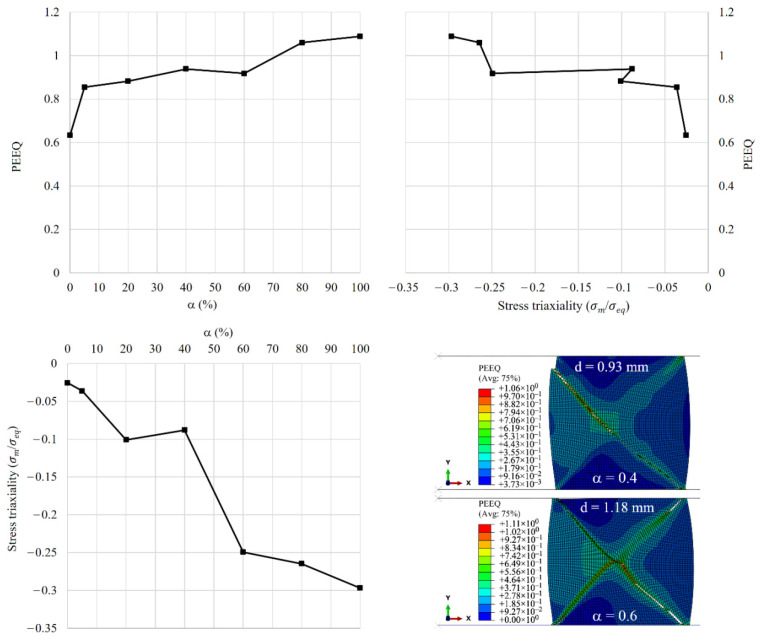
Effect of cladding ratio on stress triaxiality and equivalent plastic strain. The distribution of equivalent plastic strain is shown for the case of cladding ratio of α=0.4 at the displacement level of d=0.93 mm, and for the case of cladding ratio of α=0.6 at the displacement level of d=1.18 mm.

Figure 13 depicts the change in stress triaxiality and equivalent plastic strain (PEEQ) with different cladding ratios at the onset of crack initiation. The stress triaxiality and equivalent plastic strain are picked from the centroid of the first element deleted before crack initiation at the corners of the outer wall (i.e., at the top-right corner). As the volume fraction of the rate-sensitive material at the outer wall increases, stress triaxiality decreases and equivalent plastic strain increases. Moreover, fracture strain increases as triaxiality decreases, consistent with the experimental observations of Wierzbicki et al. [25].

### 4.3. Effects of Applied Strain Rate

**Figure 14:**
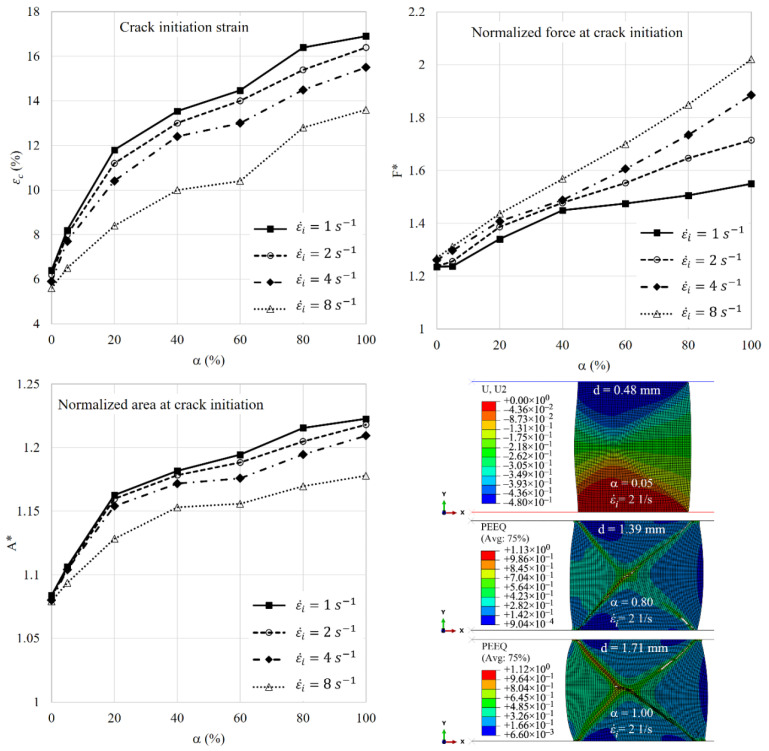
Effect of strain rate and cladding ratio on fracture parameters. The stroke of top platen, *d*, is shown at the top of the models.

The value of strain rate sensitivity parameter and magnitude of dynamic loading both play important roles in barreling and fracture retardation or advancement. Four values of applied initial strain rate, namely ${\dot{\varepsilon }}_{i}=\left[1,\;2,\;4,\;8\right]\;s^{-1}$, similar to the work of Shi et al. [11], are selected to investigate the effects of strain rate on the fracture parameters of compressed rings with different cladding ratios. The results of normalized force, normalized area and strain at crack initiation are shown in Figure 14. Increasing the applied strain rate leads to relatively low values of fracture strain, low values of the normalized area and high values of the normalized force. The influence of strain rate on the three fracture parameters becomes stronger as the cladding ratio increases (e.g., greater than 40%). The two geometrical parameters, namely crack initiation and normalized area, show a nonlinear response with respect to the cladding ratio. However, the normalized force tends to change almost linearly. Using the definition of the normalized area at crack initiation, one can conclude that barreling is lessened at higher compression speeds. Given that the slope of the non-linear curves of geometrical parameters has a transition point, as also observed in the previous section, there can be an optimum value for the cladding ratio of the rate-sensitive material, particularly at low strain rates. In the case of this study, the optimum volume fraction of the rate-sensitive material at the outer wall to enhance the ductility of the ring is about 20%. It should be emphasized that the point here is to introduce a quantitative measure based on which the optimum amount of cladding material can be selected and that the above value is the outcome of numerical analyses of the specific model of this study and can be different in other configurational set ups including different materials.

### 4.4. Fracture Strain Comparison by Shape Factor (λ)

The shape factor (λ) in a ring is related to the geometry of its cross-section and is expressed as follows:

(12) $$\lambda =\frac{2H}{OD-ID}\;.\;$$

**Figure 15:**
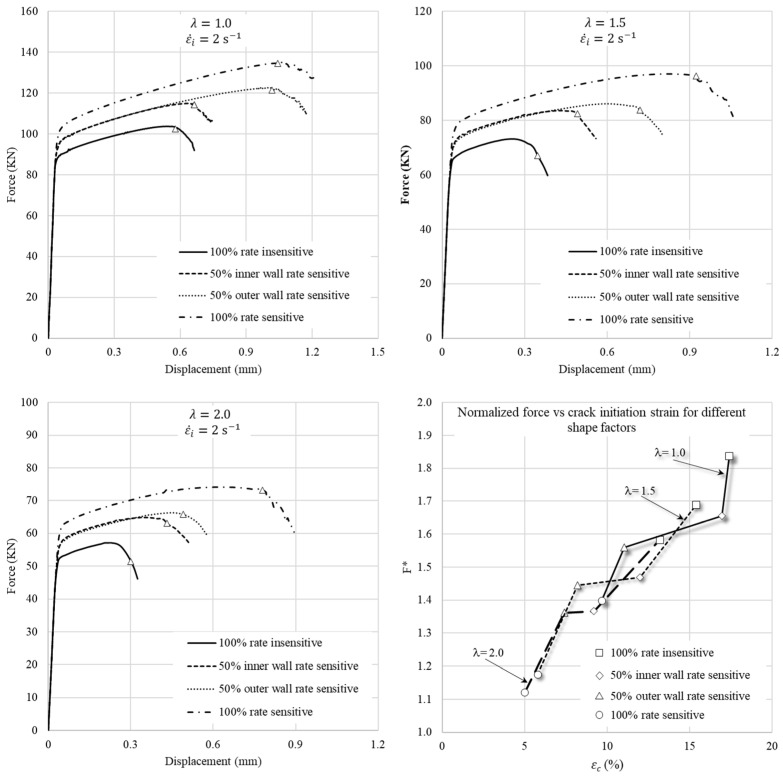
Effect of shape factor on fracture parameters.

In the previous sections, a ring with the standard geometry of 6:3:2 and OD:ID:H dimensions of 18 mm:9 mm:6 mm, resulting in a shape factor of 1.33, was used. In this section, by changing the ID, three geometries with λ=1.0  (18 mm:6 mm:6 mm), λ=1.5  (18 mm:10 mm:6 mm) and λ=2.0  (18 mm:12 mm:6 mm) are used to investigate the effects of the shape factor on a fracture in compressed rings. The volume fraction of 50% with one layer of rate-sensitive material at the outer wall is considered in this study. Figure 15 shows the f–d curves and normalized force versus fracture strains of the three models. As the shape factor increases, the values of crack initiation strains of rings with 50% volume fraction of clad and base materials become closer together. The reason is that the inner and outer walls get closer together, and the partition line of 50% volume fraction becomes closer to the middle of the cross-section of the ring. Consequently, the effect of the location of the rate-sensitive material, i.e., inner or outer wall, becomes less remarkable. Moreover, rings with the same height but higher shape factor have less normalized force at crack initiation, implying that less force per unit mass is required to fracture narrower rings.

### 4.5. Effects of Topological Interface

In the previous sections, the transition zone between the rate-sensitive and the rate-insensitive materials was modeled by a non-layered and sudden change between the materials, resulting in a one-step jump in the material characteristics. This is a common simplification that is usually considered in FEA of functionally graded materials (FGMs) [38]. To evaluate the effects of topology of the transition zone in FGM analysis, two other topologies are defined at the bonding region of the rate-sensitive and rate-insensitive materials, and their effect on the f–d curves are compared in this section. This is to investigate how the FE simulation of a fracture in compressed rings can be affected by the transition zone in the model.

**Figure 16:**
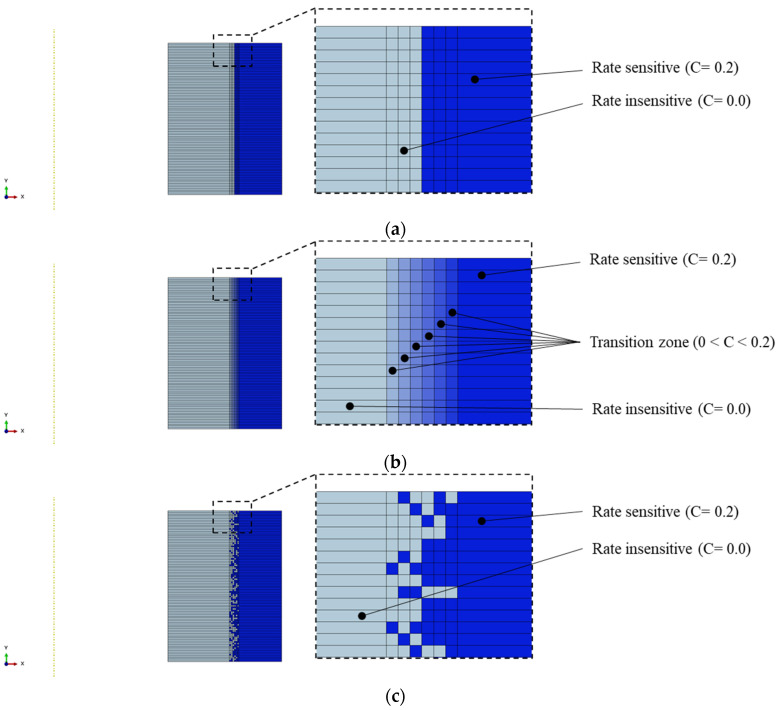
Different topologies of materials at the bonding surface of rate-sensitive and insensitive material: (**a**) non-layered, (**b**) ordered layers and (**c**) noise diffusion.

**Figure 17:**
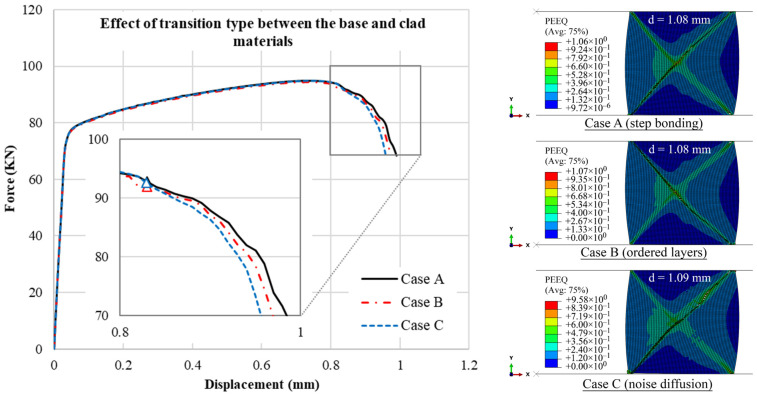
Effect of transition type on crack propagation, in terms of f–d curves and distribution of equivalent plastic strain at displacement level of: d=1.08 mm for Case A, d=1.08 mm for Case B, and d=1.09 mm for Case C.

Figure 16a shows the non-layered case, i.e., Case A, in which there is a one-step sharp interface from the base to the clad material at the partition line of 50% volume fraction. In the ordered layer model in Figure 16b, i.e., Case B, the transition from the rate-sensitive to the rate-insensitive material is simulated through stacked layers. Here, by having six additional narrow layers, the rate sensitivity parameter increases in seven steps from 0 to 0.2. The last transition model, i.e., Case C, is the noise diffusion case, as shown in Figure 16c. A MATLAB code is written to generate a random noise transition (or gradient) based on values of 0 and 1. The random values are biased in the six middle layers and gradually change to have more base material in the left three columns and more clad material in the right three columns. The values between 0 and 0.5 represent a rate-insensitive material, whereas the values between 0.5 and 1 denote a rate-sensitive material. On the basis of the location of elements along the x-direction in the model, a condition is set to keep the equal volume fraction of 50% for both materials with a minor error of $1\times 10^{-3}$. The results are shown in Figure 17. Given that the volume fraction of the clad and core materials are the same, the behavior of the f–d curves is the same in the three cases. The responses are almost the same up to the crack initiation point. The reason is that the crack starts to form from the corners of the outer wall, and in all these cases, the material is the same at the outer walls with a relatively sufficient distance with the partition line, and it cannot be influenced by the transition zone. The interface of the two different materials is also assumed to have a perfect bonding condition. The difference is seen in the crack propagation area. As the transition zone becomes smoother and more realistic from Case A to Case C, the crack grows faster in the model. Gang et al. [36] experimentally demonstrated that crack grows quickly after initiation in a compressed ring. Considering the behavior of the three models in Figure 17, the model with the noise diffusion transition zone, i.e., Case C, ruptures faster than the two other models after crack initiation, implying that Case C can provide a more realistic or suitable simulation of crack propagation in the compressed ring and FGMs. However, preprocessing the models requires additional steps. In general, there is almost no difference between Cases A, B and C before initiation of crack because the transition zone is relatively away from the corner of the outer walls where the crack starts, and the volume fraction remains the same.

### 4.6. Crack Initiation and Propagation

**Figure 18:**
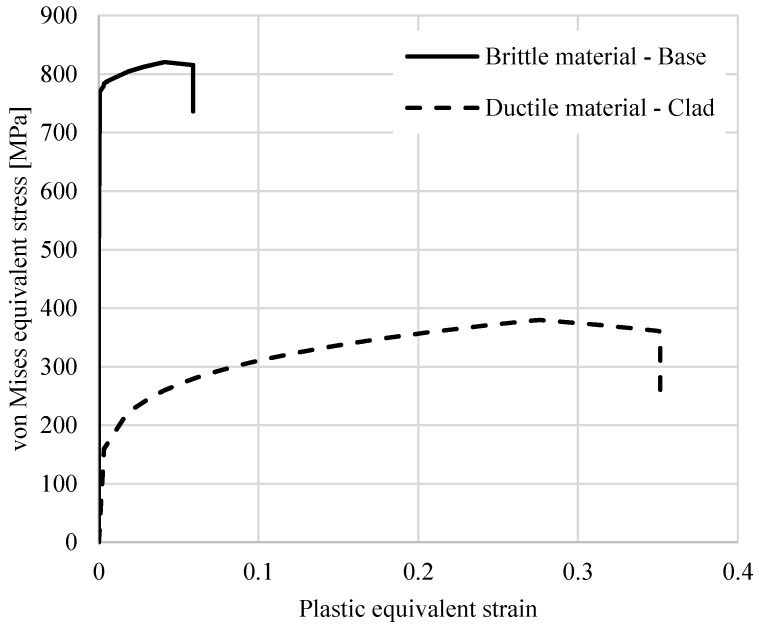
Developed brittle and ductile materials.

In the previous sections, the base and clad materials had the same material and fracture model parameters, except the parameter for strain rate sensitivity, C, in the J–C material model. An explanation could be that the location of the crack initiation remains at the corner of the outer wall in all the laminated cases similar to the case of the compressed ring made of a monolithic material, as obtained in Refs. [20,36], and that there is no change in the direction of the crack propagation path. In this section, in order to investigate the effect of material properties on crack initiation and propagation path, two completely different materials are used in the FE simulations. One of them represents a rate-independent clad material, whereas the other one represents a rate-sensitive brittle base material (Figure 18). These two materials are developed by modifying the material model and fracture model parameters in the software to exhibit similar behavior as those used in the experiments of Syn et al. [37].

The clad material uses a power law model, which is expressed as follows:

(13) $$\bar{\varepsilon }=\{\begin{array}{l} \frac{\bar{\sigma }}{E}\;\;\;\;\;\;\;\;\;\;\;\;\;\;\;\;\;\;\;\;\;for\bar{\sigma }\leq \sigma_{y}\\ \frac{\sigma_{y}}{E}{\left(\frac{\bar{\sigma }}{\sigma_{y}}\right)}^{n}\;\;\;\;\;\;\;\;\;for\bar{\sigma }>\sigma_{y} \end{array}\;$$

where $\sigma_{y}$ is the yield stress, and n is the strain hardening exponent. The clad material is soft and has a low yield stress but with a high strain hardening rate ($\sigma_{y}$ = 142 MPa, n = 5). The brittle base material is represented by the Swift law material model as follows:

(14) $$\sigma_{eq}=A+B\varepsilon_{P}^{n}=764+216\;\varepsilon_{P}^{0.42}.$$

The J–C fracture parameters of these materials are presented in Table 3. It should be noted that the presented results and the overall conclusions of this section do not specifically depend on the values of these materials’ parameters but rather on their behavior.

**Figure 19:**
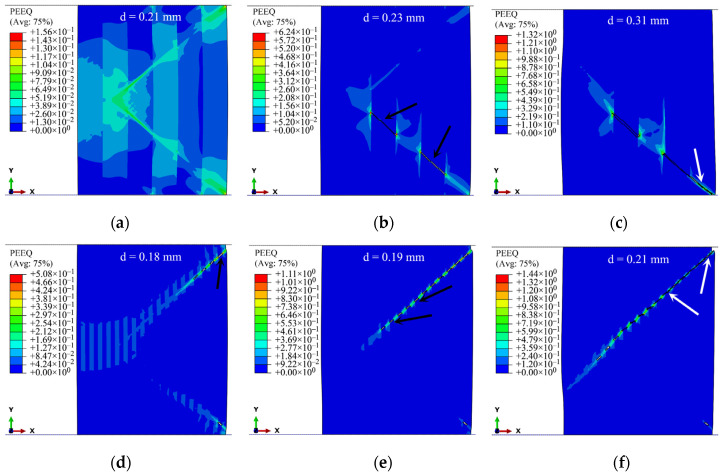
Sequence of crack initiation and growth in 3by3 and 18by18 models at ${\dot{\varepsilon }}_{i}$= 1 s^−1^: (**a**) formation of shear bands; (**b**,**d**,**e**) cracks form and grow in the layers of the brittle base material that are close to the outer wall; (**c**,**f**) layers of the ductile material start to crack from the outer wall and connect the existing cracks in the brittle layers along the direction of shear bands. The stroke of top platen, *d*, is shown at the top of the models.

The 3by3, 6by6 and 18by18 models with the layers of ductile material at the outer walls are used in this section. The sequence of crack initiation and growth in the 3by3 and 18by18 models is illustrated in Figure 19. In contrast to the rings with monolithic materials, here, the crack initiates inside the ring after the formation of shear bands. Cracks start to form in the layers of the brittle base material that are close to the outer wall along the direction of shear bands, as shown by the black arrows in Figure 19. Then, the layers of the ductile material start to crack from the outer wall and connect the existing cracks in the brittle layers along the direction of shear bands, as shown by the white arrows in Figure 19. This type of behavior, crack formation at the inner layers, was experimentally observed by Ohashi et al. [39] while studying the fracture behavior of a laminated steel–brass composite in bend tests. Their experiments showed that the relatively brittle layers of steel could fail more readily than the soft brass layers.

**Figure 20:**
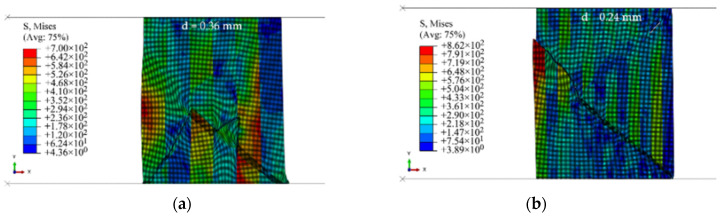
Crack propagation path in (**a**) 3by3 model at ${\dot{\varepsilon }}_{i}$ = 1 s^−1^ and displacement level of d=0.36 mm and (**b**) 6by6 model at ${\dot{\varepsilon }}_{i}$ = 4 s^−1^ and displacement level of d=0.24 mm.

**Figure 21:**
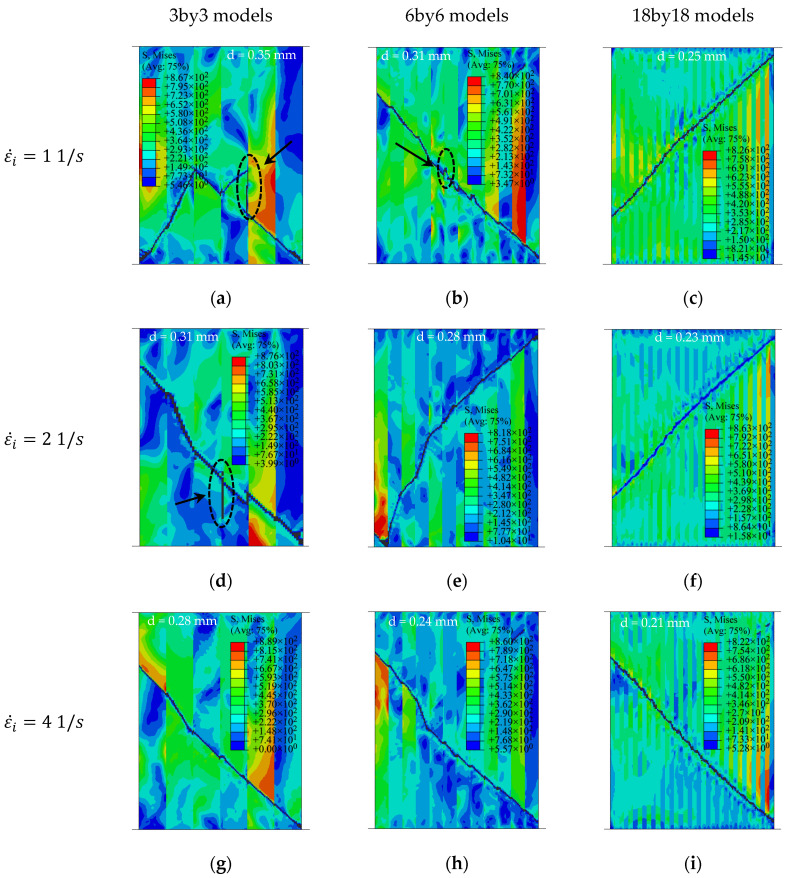
Redirection of fracture path and delamination-like behavior at the interfaces of ductile and brittle materials: (**a**) 3by3 at ${\dot{\varepsilon }}_{i}$ = 1 s^−1^, (**b**) 6by6 at ${\dot{\varepsilon }}_{i}$ = 1 s^−1^, (**c**) 18by18 at ${\dot{\varepsilon }}_{i}$ = 1 s^−1^, (**d**) 3by3 at ${\dot{\varepsilon }}_{i}$ = 2 s^−1^, (**e**) 6by6 at ${\dot{\varepsilon }}_{i}$ = 2 s^−1^, (**f**) 18by18 ${\dot{\varepsilon }}_{i}$ = 2 s^−1^, (**g**) 3by3 at ${\dot{\varepsilon }}_{i}$ = 4 s^−1^, (**h**) 6by6 at ${\dot{\varepsilon }}_{i}$ = 4 s^−1^ and (**i**) 18by18 at ${\dot{\varepsilon }}_{i}$ = 4 s^−1^. The stroke, *d*, is shown at the top of the models.

Figure 20 shows the results of the 3by3 and 6by6 models at strain rates of 1 and 4 s^−1^, respectively. The volume fractions of base–clad materials for the three cases of 3by3, 6by6, and 18by18 models are approximately 47–53%, 49–51%, and 50–50%, respectively. In the case of the 3by3 model at ${\dot{\varepsilon }}_{i}$ = 1 s^−1^ (Figure 20a), the crack starts to form from the inside of the model at the brittle layers, as also shown in Figure 19b. Here, the crack does not grow along a straight line, and it changes its direction. For better illustration, the deleted elements are shown on discrete banded contour plots of undeformed cross-sections (Figure 21).

The right side of the cross-sections presents the outer wall of the rings. The direction of the fracture path is a function of the thickness of layers. Delamination-like behavior or crack branching occurs at the interface in the 3by3 model with a sharp change in the angle of the crack path at lower strain rates, as shown in Figure 21a. Similar behavior is observed in the 6by6 model (Figure 21b). As the strain rate increases, less delamination and change in the direction of the fracture path is observed (Figure 21c,g). The 18by18 models do not experience delamination or sudden change in the fracture path, as they have thinner layers. These behaviors are similar to the experimental observations of Syn et al. [37] for tensile specimens. The numerical results of this work show that delamination could occur even in the presence of perfect bonding conditions due to the high difference between the material and fracture behavior of laminated layers at low strain rates. As the strain rate increases, the fracture path becomes slightly straighter in the 18by18 models.

**Figure 22:**
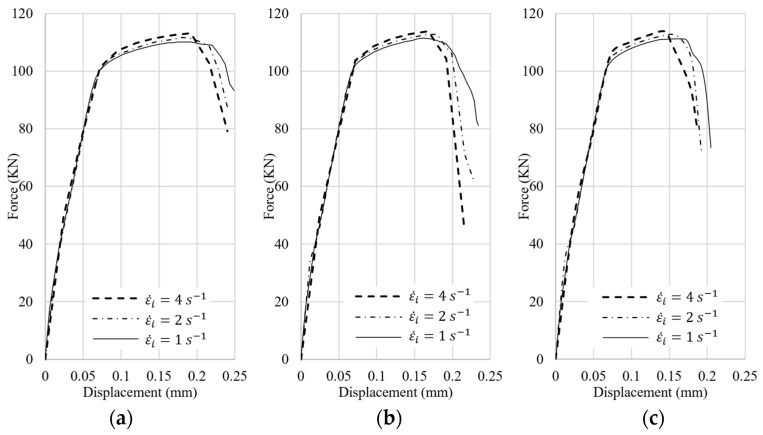
Force–displacement curves of (**a**) 3by3 models, (**b**) 6by6 models and (**c**) 18by18 models.

The force–displacement curves of these nine cases are shown in Figure 22. For the cases of 3by3 models, Figure 22a, where the thickness of the outer layer is more compared to the other two cases, the behavior of the curves is more similar to that of the clad material with more ductility. The volume fraction of clad material is 53% in the 3by3 models, whereas it is around 50% in the 18by18 models, resulting in less ductility in the 18by18 models. As the strain rate increases, the crack initiates sooner. It should be emphasized that the presented results and conclusions in this section are based on the numerical simulations with the two materials that are developed by modifying the material model and fracture model parameters in the software for this study and can be different for different material models and configurations.

**Figure 23:**
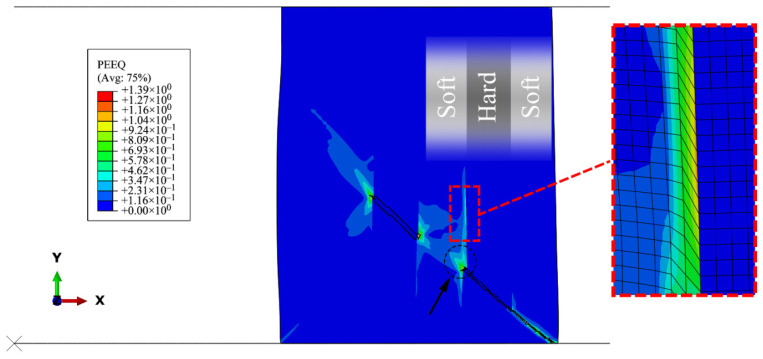
Secondary shear band at the interface of soft and hard materials in the 3by3 model before fracture, at the displacement level of d=0.31 mm.

Figure 23 shows the location of crack branching in the 3by3 model at ${\dot{\varepsilon }}_{i}$ = 1 s^−1^ before fracture. The detailed deformation of the elements at the interfaces shows that a secondary shear zone is formed vertically at the crack tip in the ductile material. The relatively high layer thickness does not allow the accumulation of plastic strain at the crack tips to connect the cracks along the primary shear bands. As the layer thickness decreases or as the strain rate increases, this phenomenon is observed less, resulting in less change in the direction of the fracture path. When the distance between the layers is relatively low, less time is available for the secondary shear band to be formed, and the crack tips are connected more quickly.

## 5. Conclusions

ABAQUS/Explicit package and the Johnson-Cook failure model were used to study the topological effects of cladding strain rate-sensitive and rate-insensitive layers on the ductility and fracture of rings under axial compression. This numerical study has led to the following conclusions:

1. The f–d curves of cladded rings with a different number of layers of rate-sensitive and rate-insensitive material were located between the f–d curves of compressed rings with monolithic material. While the outer wall had a rate-sensitive material, ductility increased by about 25%, when the number of layers decreased from 18 by 18 to 1 by 1.
2. Ductility was found to be governed by the behavior of the material located at the outer wall regardless of the volume fraction. This is because crack initiates from the corner of the outer walls. However, as the thickness of the layers decreased and their numbers increased, the difference between having a rate-sensitive or rate-insensitive material at the outer wall was observed less (i.e., the f–d curves became closer to each other).
3. The amount of barreling at the outer wall was found to have a direct relationship with ductility. More barreling at the outer wall resulted in higher fracture strain. On the other hand, the advancement in the fracture can be associated with more barreling on the inner surface. Similar to the effect of friction in the ring compression test, the direction of material flow can be affected by material parameters, including strain rate sensitivity parameter as well as the magnitude of dynamic loading. Less barreling was observed when the compression speed increased.
4. The effects of the shape factor on the fracture of the compressed rings were evaluated by changing the inner radius. As the shape factor increased, the role of the location of the rate-sensitive material became less remarkable. Moreover, fracture occurred at less force per unit volume for the narrower rings.
5. The effect of the topology of the material at the interface of rate-sensitive and rate-insensitive materials in the ring was investigated. Differences in bonding types (e.g., layered or noise diffusion) did not substantially affect the crack initiation because the interface was away from the outer corners. However, crack propagation was quicker in the case of the noise diffusion interface. This behavior may be more suitable in numerical simulations of the interface of FGMs, as it was also experimentally observed by Gang et al. [36] that crack grows quickly across compressed rings.
6. Delamination-like behavior or crack branching can be a function of layer thickness and strain rate. As layer thickness increased, more delamination or crack branching was observed at the interface of the laminated layers. Furthermore, at high strain rates, less delamination and change in the direction of the fracture path was observed.

## Figures and Tables

**Table 1:** The J–C material model parameters [34].

*A* (MPa)	*B* (MPa)	*n*	*C*	*m*	${\dot{\varepsilon }}_{0}(1/s)$	*T_m_* (K)	*T_0_* (K)
324	114	0.42	0.002	1.34	1.0	925	293.2

**Table 2:** The input fracture parameters for the J–C model [34].

*D*1	*D*2	*D*3	*D*4	*D*5
−0.77	1.45	−0.47	0.00	1.60

**Table 3:** The J–C failure model parameters for the developed materials.

	*D*1	*D*2	*D*3	*D*4	*D*5
Clad	−1.77	0.45	1.47	0.00	1.60
Base	−1.77	1.42	0.49	0.00	1.60

## Data Availability

Data sharing is not applicable.
